# Sigma-1 Receptor Is Critical for Mitochondrial Activity and Unfolded Protein Response in Larval Zebrafish

**DOI:** 10.3390/ijms222011049

**Published:** 2021-10-13

**Authors:** Lucie Crouzier, Morgane Denus, Elodie M. Richard, Amarande Tavernier, Camille Diez, Nicolas Cubedo, Tangui Maurice, Benjamin Delprat

**Affiliations:** MMDN, University of Montpellier, EPHE, INSERM, 34095 Montpellier, France; lucie.crouzier@umontpellier.fr (L.C.); morgane.denus@umontpellier.fr (M.D.); elodie.richard@umontpellier.fr (E.M.R.); amarande.tavernier@etu.umontpellier.fr (A.T.); camille.diez@inserm.fr (C.D.); nicolas.cubedo@inserm.fr (N.C.); tangui.maurice@umontpellier.fr (T.M.)

**Keywords:** sigma-1 receptor, phenotyping, zebrafish, ER stress, mitochondria bioenergetics

## Abstract

The sigma-1 receptor (S1R) is a highly conserved transmembrane protein highly enriched in mitochondria-associated endoplasmic reticulum (ER) membranes, where it interacts with several partners involved in ER-mitochondria Ca^2+^ transfer, activation of the ER stress pathways, and mitochondria function. We characterized a new S1R deficient zebrafish line and analyzed the impact of S1R deficiency on visual, auditory and locomotor functions. The *s1r^+25/+25^* mutant line showed impairments in visual and locomotor functions compared to *s1r^WT^*. The locomotion of the *s1r^+25/+25^* larvae, at 5 days post fertilization, was increased in the light and dark phases of the visual motor response. No deficit was observed in acoustic startle response. A critical role of S1R was shown in ER stress pathways and mitochondrial activity. Using qPCR to analyze the unfolded protein response genes, we observed that loss of S1R led to decreased levels of IRE1 and PERK-related effectors and increased over-expression of most of the effectors after a tunicamycin challenge. Finally, S1R deficiency led to alterations in mitochondria bioenergetics with decreased in basal, ATP-linked and non-mitochondrial respiration and following tunicamycin challenge. In conclusion, this new zebrafish model confirmed the importance of S1R activity on ER-mitochondria communication. It will be a useful tool to further analyze the physiopathological roles of S1R.

## 1. Introduction

The σ_1_ receptor (S1R) is a transmembrane protein (25 kDa) of the endoplasmic reticulum (ER), particularly enriched at the mitochondria-associated ER membrane (MAM) [1,2]. In physiological conditions, its primary function is to act as a signal modulator that chaperones different partner proteins including inositol 1,4,5-trisphosphate receptor (IP3R), glucose-regulated protein (GRP-78; BiP), or inositol-requiring enzyme 1 (IRE1), among others [1,3,4,5], leading to a modulation of several cellular responses and signaling pathways [6]. In cellular stress conditions, S1R dissociates from BiP, modulates cellular Ca^2+^ homeostasis from ER to mitochondria through different mechanisms, impacting a variety of intracellular signal transduction systems [7,8,9]. Interestingly, *S1R* is expressed in different tissues such as the central nervous system (hippocampus, frontal cortex and olfactory bulb), heart, lungs, and kidneys, as well as endocrine, immune and reproductive tissues [10,11,12]. 

S1R activity can be triggered by small molecules acting as S1R agonists. These molecules have shown therapeutic effects, which were reported to decrease pathological phenotypes in neurodegenerative disorders (for reviews, see [13,14]) including Alzheimer’s disease, Parkinson’s disease, Huntington’s disease and amyotrophic lateral sclerosis (ALS) [15,16,17]. The curative effects were also measured in other diseases such as cardiac disorder [18,19], chronic pain [20,21], depression [22,23,24], addiction [25,26] and cancer [11,27].

In addition, genetic alterations of the *SIGMAR1* gene have been associated with severe neurodegenerative disorders. Several truncations/deletions or point mutations in *SIGMAR1* generated distal hereditary motor neuropathy [28,29], while juvenile cases of ALS were associated with a missense mutation (c.304G>C, p.E102Q) and a frameshift mutation (c.283dupC, p.L95fs) in the *SIGMAR1* gene sequence [30,31]. Moreover, in mouse models, the absence of S1R induced mild developmental damages. Indeed, S1R knockout (KO) mouse models presented a loss of retinal cells particularly in the ganglion cell layer, axonal degeneration of the optic nerve head, an abnormal electroretinogram, decreased concentration of mature brain-derived neurotrophic factor (BDNF), alterations in motor response, alterations in hippocampal cells, abnormal heart-brain axis responses, basal ganglia damages, progressive systolic cardiac dysfunction, oxidative stress in the liver, increased somatosensitivity, muscle and motoneuron axons strength decrease at the neuromuscular junction, alteration of BDNF retrograde transport velocity in motoneurons, cognitive and psychiatric decrements, and increased cell death [32]. Collectively, these data strengthen the importance of S1R in brain development and in the etiology of neurodegenerative disorders. This also fosters the importance of developing complementary animal models that will allow screening small molecules activating (such as PRE-084, ANAVEX2-73, Pridopidine, Cutamesine) or inactivating (such as NE-100, S1RA) S1R and also to help better understand its in vivo physiopathological role in many of these disorders.

The zebrafish has proven an efficient animal model for research on human diseases, particularly for neurological and neurodegenerative disorders [33,34,35,36]. The zebrafish presents numerous valuable advantages for pharmacological and toxicological research, including the fact that 70% of human protein-coding genes are linked to genes found in fish and 84% of genes known to be associated with human disease have homologous genes [37], but also it benefits from simple husbandry and high fecundity, simplicity of generating offspring, a small size and rapid development [38]. A zebrafish line with inactivated S1R was recently created, named *s1r^+25/+25^* [39]. We characterized the physiological impacts of S1R invalidation in *s1r^+25/+25^* zebrafish larvae, and different phenotypic parameters were measured including neurosensory functions (vision, hearing), locomotion, morphological alterations and metabolism. Mutant *s1r^+25/+25^* zebrafish larvae presented a marked locomotor alteration and, at the physiological level, alterations in ER stress response as well as abnormal mitochondrial respiration. This study confirmed in a newly developed S1R KO zebrafish model, the importance of S1R as a MAMs resident protein, importantly modulating ER-mitochondrial communication.

## 2. Results

### 2.1. Characterization of the s1r^+25/+25^ Mutant Zebrafish Line

**Figure 1:**
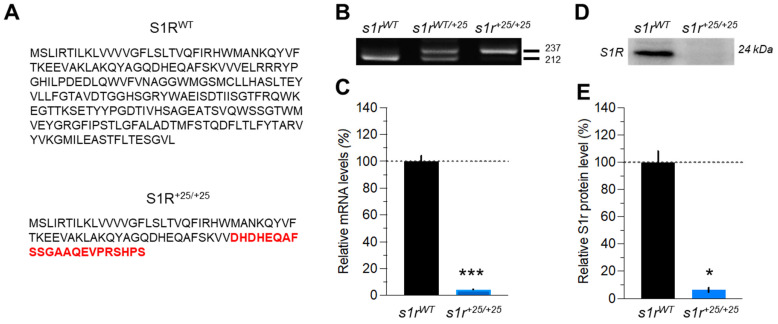
Characterization of the CRISPR/cas9-induced *s1r* mutant zebrafish line. (**A**) Amino acids sequence of the S1R protein with or without mutation. New inserted amino acids are depicted in red. (**B**) A targeted fragment was amplified by PCR from the genomic DNA of adult zebrafish tail and analyzed after migration on agarose gel. (**C**) Relative *sigmar1* mRNA level revealed by qPCR in *s1r^+25/+25^* and *s1r^WT^* zebrafish larvae. Expression of *sigmar1* was normalized using the *zef1α* reference gene. (**D**) Representative Western blot of whole lysates from wild-type (*s1r^WT^*) or homozygous *s1r^+25/+25^* mutant zebrafish. (**E**) Relative S1R protein level in the whole larvae at 5 dpf, normalized using total protein expression in the samples (Stain-Free^™^). Data are shown as the mean ± SEM from *n* = 12 per group in (**C**) and *n* = 4 in (**E**). * *p* < 0.05, *** *p* < 0.001 vs. *s1r^WT^* controls; Man n-Whitney’s test.

S1R was firstly described as a protein with two putative transmembrane domains and two hydrophobic domains [1,40,41]. Its N-ter and C-ter ends, the latter possessing chaperone activity, were predicted to be expressed in the ER lumen. Nevertheless, other groups have suggested a one transmembrane domain with a N-ter luminal and C-ter cytoplasmic localization [42]. Mavlyutov et al. [43] and Zhemkov et al. [9] proposed a one transmembrane domain with a N-ter cytosolic and C-ter luminal localization. As of today, no consensus topology of the protein has been clearly established. The sequence drawn in red shows the amino acids representing new, erroneous, residues predicted to occur after the reading frame shift, followed by a premature stop codon in the C-ter region (Figure 1A). Using CRISPR/Cas9-mediated mutagenesis, Rennekamp et al. [39] generated a zebrafish line with a 25 bp insertion in exon 2 of the *sigmar1* gene, leading to a shift of the reading frame. The mutation was created just upstream of the gene sequence that encodes the most conserved part of the S1R protein. PCR confirmed that the *s1r* mutants had additional 25 bp in the coding region, thus showing a single band of 237 nucleotides compared to control larvae 212 nucleotides band (Figure 1B). Mutation was predicted to result in loss of protein function due to the frameshift and the introduction of an early stop codon.

Disruption of both gene and protein expressions were confirmed by quantitative PCR and Western blot analyses. First, qPCR analysis showed that larvae carrying the mutation had a decreased *sigmar1* expression level compared to control larvae, confirming that the mutation induced mRNA decay (Figure 1C). Second, Western blotting (Figure 1D) and its quantification (Figure 1E) revealed a drastic decrease of S1R expression in *s1r^+25/+25^* fish compared to controls, validating this zebrafish line as a S1R KO model.

Mutant homozygous larvae did not show any overt morphological abnormalities in terms of body size, eye diameter, ear area and anterior and posterior otoliths (Supplementary Figure S1). They grew and became viable and fertile adults. These observations incidentally confirmed that no gross morphological alterations would likely compromise the subsequent behavioral analyses. 

### 2.2. The s1r^+25/+25^ Larvae Exhibited Increased Locomotor Response

**Figure 2:**
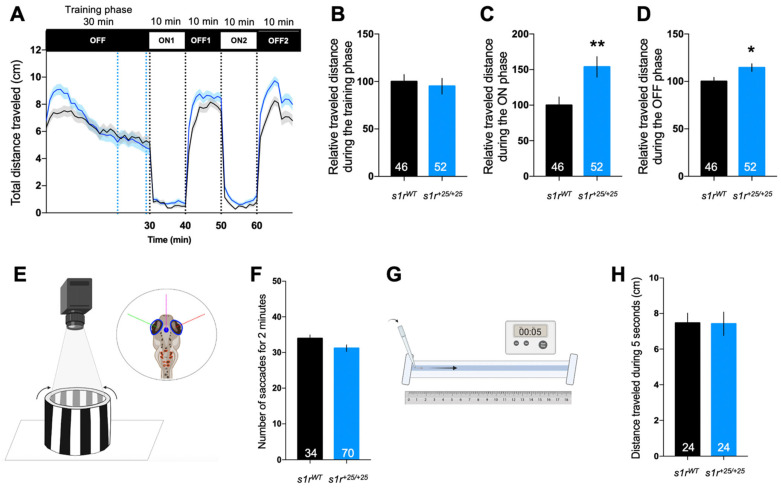
Behavioral analyses of 5 dpf *s1r* mutant larvae. (**A**) Analysis of the distance traveled by the larvae during the light/dark (intensity 100%/0%) cycle in the VMR test. The activity was measured for 70 min with a training phase of 30 min in the dark (OFF 0%), then two cycles of light/dark (ON 100%/OFF 0%) of 10 min each. The graph shows the distance traveled per min for *s1r^WT^* and *s1r^+25/+25^* larvae. (**B**) Relative distance traveled during the training phase [blue dotted lines in (**C**), between 21 and 29 min]; (**A**) during the ON phase [the averaged ON1 and ON2 phases]; (**D**) during the OFF phase [the averaged OFF1 and OFF2 phases]. Relative distance was expressed as % of controls. (**E**) Illustration of the OKR test. (**F**) Quantification of the number of saccades performed in 2 min. (**G**) Illustration of the touch escape test. The larva was placed in a rail filled with E3 medium, the tail was touched with a tip and the distance was measured. (**H**) The distance traveled was measured during 5 s, repeated three times per larva and averaged. Data show mean ± SEM from three replicas. The number of animals is indicated within the columns. * *p* < 0.05, ** *p* < 0.01; unpaired *t*-test.

As S1R has been shown to have a role in locomotion [32,44], we first assessed the impact of S1R invalidation on global locomotor activity of the mutant larvae using the visual motor response (VMR) assay. Larvae were placed in individual wells of a 96-well plate and their activity, following sudden changes in light intensity from 100% (ON) to 0% (OFF), was recorded during the whole experiment (Figure 2A). The distance travelled during the training phase did not differ between control and mutant fish (Figure 2B). However, significant increases in locomotion were measured for the *s1r^+25/+25^* larvae during both the ON and OFF periods (Figure 2C,D).

The optokinetic response (OKR) assay allowed us to directly measure visual acuity of the larva by immobilizing them in a methylcellulose solution placed at the center of a cylinder on which black-and-white strips were rotating (Figure 2E). Analysis of the eye movements showed no difference in the number of saccades between mutant and control larvae at 5 dpf (Figure 2F). The touch-escape response, measuring a reflex-driven locomotor response, was also measured (Figure 2G). The distance traveled during 5 s after the tactile stimulation was similar between mutant and control larvae (Figure 2H). These observations, therefore, indicated that S1R KO zebrafish did present locomotor alteration in the VMR test that is unrelated to visual deficit or spontaneous hyperactivity. 

**Figure 3:**
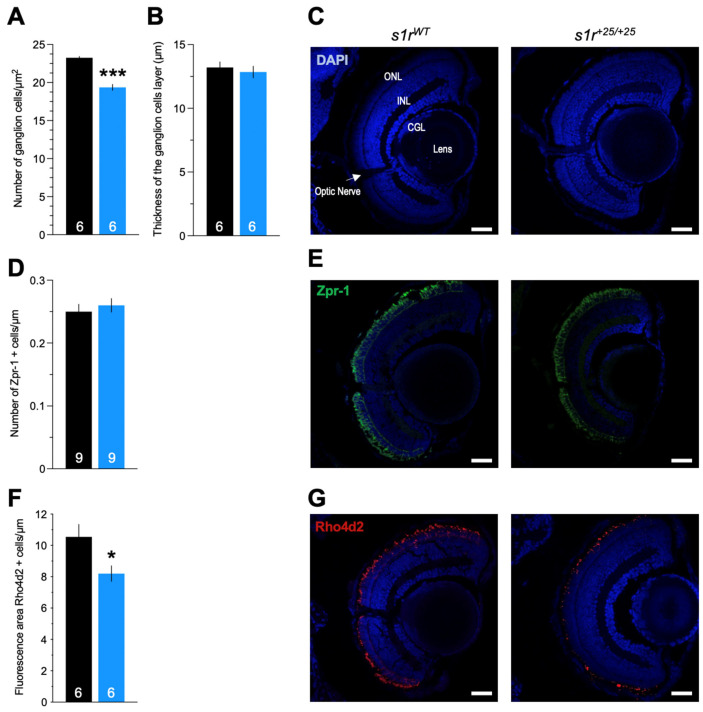
Morphological analysis of the neurons in the zebrafish retina. (**A**) Quantification of the number of ganglion cells, (**B**) quantification of the thickness of the associated layer, and (**C**) typical micrographs of the retina. Confocal images were obtained from section from *s1r^WT^* and *s1r^+25/+25^* zebrafish retina, showing cell nuclei labeled with 4′,6-diamidino-2-phenylindole (DAPI, blue). (**D**) Quantification of photoreceptor cells (red and green cones) labelled with Zpr-1 antibody and (**E**) typical micrographs of the cones (green). (**F**) Quantification of rods labelled with Rho4d2 antibody and (**G**) typical micrographs of the rods (red). Abbreviations: GCL, ganglion cell layer; INL, inner nuclear layer; ONL, outer nuclear layer. Scale bars in (**C**,**E**,**G**) = 30 µm. The number of animals is indicated in the columns. * *p* < 0.05, *** *p* < 0.001; unpaired *t*-test.

We performed an immunohistochemical analysis of eye sections from both *s1r^WT^* and *s1r^+25/+25^* larvae and observed no grossly observable nor specific morphological abnormalities of ganglion cells (Figure 3A–C), and red and green cones labeled using Zpr-1 antibody (Figure 3D,E). Interestingly, the number of rods, labeled using Rho4d2 antibody, decreased significantly in *s1r^+25/+25^* larvae compare to controls (Figure 3F,G). The constitutive invalidation of S1R in the *s1r^+25/+25^* zebrafish resulted in an impairment of the retinal development, especially in rod cells.

Despite a lack of evidence for a role of S1R on auditory mechanisms, a study previously suggested that the selective S1R agonist cutamesine protected mouse hearing from severe noise trauma [45]. Consequently, to rule out any potential neurosensorial deficits, we tested the hearing ability of *s1r^+25/+25^* mutant larvae. Analysis of the quantity of movement of the larvae in the acoustic startle response (ASR) assay was measured at 5 dpf (Supplementary Figure S2A). Movement during training and baseline phases (Supplementary Figure S2B,C) increased significantly in *s1r^+25/+25^* larvae compared to the controls. In a coherent manner but in a different assay, hyperlocomotion was observed in the ASR assay. However, S1R invalidation had no impact on quantity of movement after the different stimulations, which suggests that the invalidation of S1R did not result in an impairment of ear development (Supplementary Figure S2D).

### 2.3. S1R Invalidation Modulated the Expression of ER Stress Genes, in Resting and Tunicamycin-Induced Pathological Conditions

**Figure 4:**
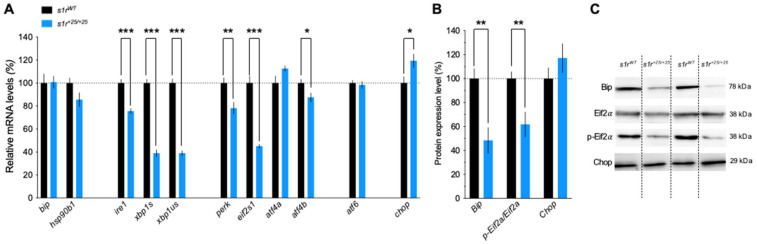
Related mRNA and protein expression levels of ER stress markers in zebrafish larvae in physiological conditions at 5 dpf. (**A**) mRNA levels were analyzed by qPCR in whole larvae. (**B**) Protein contents were analyzed by Western blot. (**C**) Representative Western blots. *zeif2α* level and Stain-Free^™^ were used as a loading control in qPCR and Western blot analyses, respectively. Data are expressed as mean ± SEM, normalized to *s1r^WT^* control level with *n* = 5–9 in each group. * *p* < 0.05, ** *p* < 0.01, *** *p* < 0.001; unpaired *t*-test.

**Figure 5:**
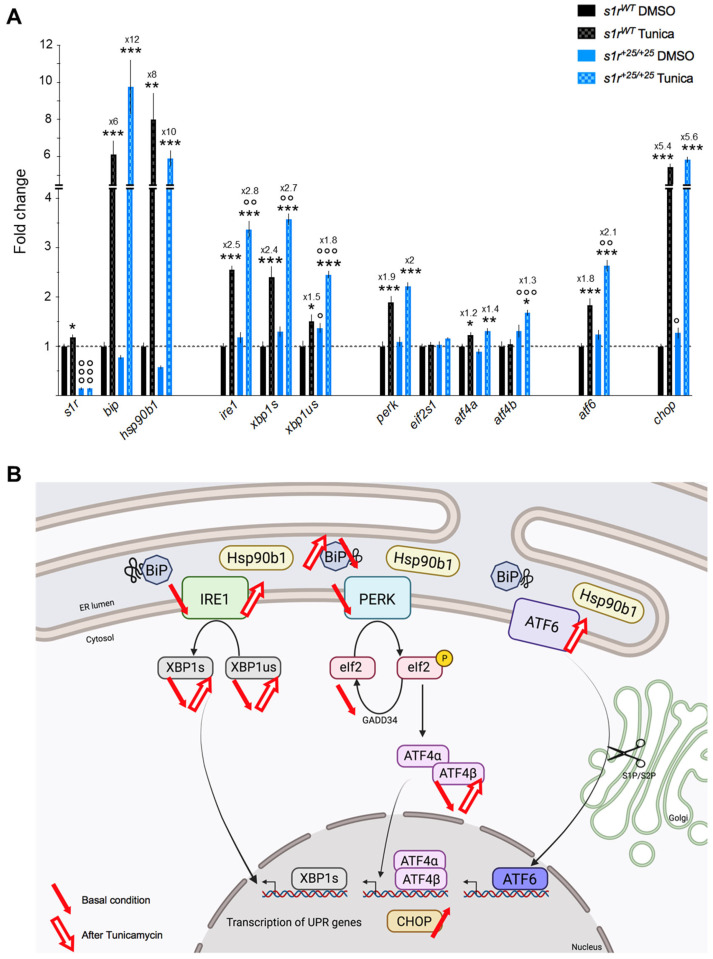
(**A**) Effect of the Tunicamycin (2 µg/mL, 24 h) challenge on ER stress gene expression levels in *s1r^+25/+25^* zebrafish larvae at 5 dpf. The selected genes were analyzed using cDNA prepared from whole zebrafish larvae and relative mRNA expression were expressed as percentage of *s1r^WT^* + 0.1% DMSO controls. The fold change from 0.1% DMSO treatment is indicated. Data are expressed in mean ± SEM of *n* = 5 determination in each group. * *p* < 0.05, ** *p* < 0.01, *** *p* < 0.001 vs. DMSO treatment; ° *p* < 0.05, °° *p* < 0.01, °°° *p* < 0.001 vs. *s1r^WT^* line; two-way ANOVA followed by unpaired *t*-test. (**B**) Schematic summary of ER stress pathway alterations observed in basal condition or after tunicamycin treatment in *s1r^+25/+25^* zebrafish line.

S1R is a key player in the ER stress response, acting as a modulator for the induction of ER stress pathways [4,9,46,47,48]. To investigate if S1R impacts ER stress modulation, we analyzed the mRNA expression levels of ER stress factor genes in the basal condition (Figure 4) and after a tunicamycin challenge (Figure 5A) in the whole zebrafish larvae. Variations are summarized in Figure 5B. 

In the control condition, the expression levels of the unfolded protein response (UPR) inducers, *bip* and *hsp90b1*, were unaffected in mutant larvae compared to controls. However, the expression levels of a large number of the UPR primary effectors related to the IRE1 and PERK pathways were consistently down-regulated in mutant larvae compared to controls. First, *ire1* and its secondary effectors, *xbp1s* and *xbp1us*, were highly significantly down-expressed (Figure 4A). Second, *perk* and its secondary effectors *eif2s1* and *atf4β* were also significantly down-regulated. Third, *atf6* was unchanged and *chop* mildly but significantly over-expressed (Figure 4A). At the protein level, Western blot analyses were done using the few specific antibodies available in zebrafish and showed that the level of Bip and p-Eif2α/Eif2α were significantly decreased in *s1r^+25/+25^* larvae compared to controls, while Chop level was unchanged (Figure 4B,C).

These results suggested that S1R invalidation in zebrafish could lead to a deregulation of the ER stress response as it impacted the expression level of numerous effectors in basal condition particularly related to the IRE1 and PERK pathways. Tunicamycin, by provoking the accumulation of unfolded proteins, is a strong ER stress inducer and is commonly used as a cellular stressor [49]. When *s1r^WT^* or *s1r^+25/+25^* larvae were treated at 4 dpf with 2 μg/mL of tunicamycin for 24 h, a strong ER stress was monitored (Figure 5A). The mRNA levels of the different markers were drastically increased in mutant and control larvae. First, *s1r* was moderately but significantly increased after tunicamycin in *s1r^WT^* larvae and, as expected, absent in *s1r^+25/+25^* larvae (Figure 5A). Second, the levels of the UPR inductors *bip* and *hsp90b1* were highly significantly increased. Third, all three UPR pathways were also mobilized with significant increases of *ire1* and its effectors *xbp1s* and *xbp1us, perk* and its effectors *atf4α* and *atf4β*, and *atf6* (Figure 5A). Finally, *chop* level was highly significantly increased (Figure 5A) as well.

Interestingly, the genotype had a major impact on the ER stress response to tunicamycin treatment, with increased over-expression of *bip, ire1, xbp1s, xbp1us*, *atf4β*, and *atf6*, in *s1r^+25/+25^* larvae as compared to *s1r^WT^* larvae (Figure 5A,B). These observations, decreased levels of IRE1 and PERK effectors in basal condition and increased over-expression of not only IRE1 and PERK effectors but also ATF6 in the ER stress condition, suggests that S1R invalidation altered the UPR response and regulation of ER stress. Interestingly, the alteration is coherent with a compensatory over-reaction of the pathways, resulting in a mild impact on Chop level.

### 2.4. S1R Invalidation Altered Mitochondrial Bioenergetics

**Figure 6:**
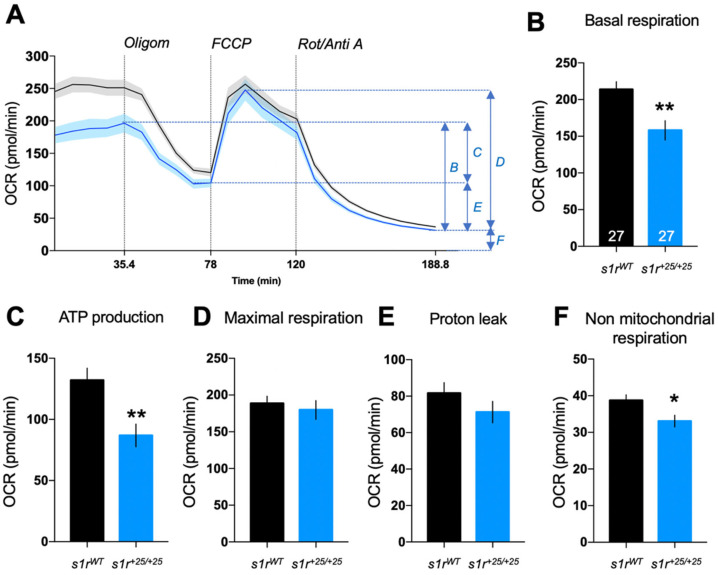
Analysis of mitochondrial respiration by the zebrafish larvae at 5 dpf analyzed using the Seahorse XF mito stress test. (**A**) Seahorse diagram depicting profile of oxygen consumption rate (OCR) during the assay. Letters (**B**–**F**) indicate the OCR components detailed in panels (**B**–**F**). (**B**) Basal respiration, (**C**) ATP production, (**D**) maximal respiration, (**E**) proton leak, (**F**) non-mitochondrial respiration. Data are shown as mean ± SEM from the number of larvae indicated in (**B**). Abbreviations: Oligom, oligomycin (25 μM); FCCP, carbonyl cyanide-p-trifluoromethoxyphenylhydrazone (8 μM); Rot/Anti A, rotenone + antimycin A (15 μM). * *p* < 0.05, ** *p* < 0.01; unpaired *t*-test.

S1R is enriched in MAMs [1] and facilitates Ca^2+^ flux from the ER to the mitochondria [1,3]. Recent studies have shown that an alteration in the cross-talk between these two organelles affects the bioenergetics of the cells [46,50,51,52]. Moreover, S1R activity directly impacts mitochondrial oxidative respiration [53]. We hypothesized that the absence of S1R would disturb the mitochondrial activity in the mutant larvae. We therefore measured the oxygen consumption rate (OCR) before and after addition of complex inhibitors or decoupling agents of the respiratory chain, namely oligomycin, FCCP, antimycin A and rotenone, and analyzed several parameters of the mitochondrial respiration (Figure 6A). Basal respiratory rate, ATP production-related and non-mitochondrial respiration (Figure 6B,C,F) were significantly decreased in *s1r^+25/+25^* mutant larvae at 5 dpf, compared to control larvae, while the maximal respiration rate and proton leak remained unchanged (Figure 6D,E). These results confirmed in the zebrafish line a specific role of S1R on bioenergetics and particularly ATP production-related mitochondrial oxidative respiration.

**Figure 7:**
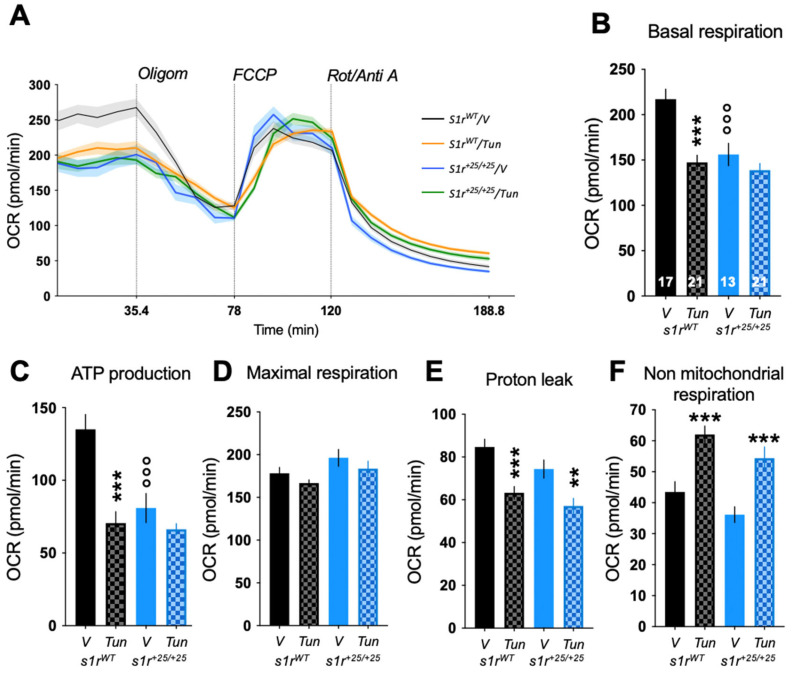
Effect of the Tunicamycin (Tun, 2 µg/mL, 24 h) challenge on mitochondrial respiration of the zebrafish larvae at 5 dpf analyzed using the Seahorse XF mito stress test. (**A**) Profiles of oxygen consumption rate (OCR) during the assay. (**B**) Basal respiration, (**C**) ATP production, (**D**) maximal respiration, (**E**) proton leak, (**F**) non-mitochondrial respiration. Data are shown as mean ± SEM from the number of larvae indicated in (**B**). Abbreviations: Oligom, oligomycin (25 μM); FCCP, carbonyl cyanide-p-trifluoromethoxyphenylhydrazone (8 μM); Rot/Anti A, rotenone + antimycin A (15 μM). ** *p* < 0.01, *** *p* < 0.001 vs. V-treated; °°° *p* < 0.001 vs. *s1r^WT^*; Newman-Keuls’ test after a two-way ANOVA.

When *s1r^WT^* or *s1r^+25/+25^* larvae were treated at 4 dpf with 2 μg/mL of tunicamycin for 24 h, mitochondrial metabolism was affected (Figure 7A). Basal respiration, ATP production and proton leak were highly decreased in *s1r^WT^* (Figure 7B,C,E), showing an impact of ER stress on mitochondrial respiration. Non-mitochondrial respiration was increased while maximal respiratory capacity was unchanged by tunicamycin in *s1r^WT^* (Figure 7D,F). Interestingly, the ER stressor failed to decrease basal respiration and ATP production-related respiration in *s1r^+25/+25^* larvae below the level measured in V-treated controls (Figure 7B,C). Maximal respiration, proton leak and non-mitochondrial respiration levels were equally affected in *s1r^WT^* and *s1r^+25/+25^* lines (Figure 7D–F), showing a S1R-dependent impact of ER stress on mitochondrial energetics. 

## 3. Discussion

In the present study we characterized a novel zebrafish line carrying a mutated S1R protein. The mutation resulted in the loss of the protein C-terminal tail, known to be carrying the activity [1]. The *s1r^+25/+25^* zebrafish line is therefore constitutively devoid of the S1R chaperone activity that allows a pleiotropic modulatory action on several intracellular pathways and interorganelle dialogs [4,49]. Our first observation was that the *s1r^+25/+25^* zebrafish developed from the larval to the adult stage with no grossly observable morphologic or functional defects. Moreover, the fish reproduced well under homozygous breeding, confirming an absence of major neuroendocrine or physiological alteration. A similar observation was made in murine KO models. Both the *Sigmar1^tm1Lmon^* mouse line developed by Esteve [44] and the *Sigmar1^Gt(IRESBetageo)33Lex^* line developed by Lexicon [54,55] did not show developmental defects and reproduced normally. The availability of a S1R KO zebrafish line will be of major use for future research, particularly since: (i) zebrafish embryos are nearly transparent which allows easy examination of the development of internal structures; (ii) the model is particularly prone to in vivo imaging and electrophysiological approaches; and (iii) eggs are fertilized and developed outside the mother’s body, which makes zebrafish an ideal model organism for studying early development [56]. Indeed, S1R may play a major yet underexplored role during early development of many organs, including the brain. For instance, S1R was shown to regulate the formation of dendritic spines in hippocampal neurons and the knockdown of S1R by siRNAs provoked a deficit in the formation of dendritic spines [57,58]. Moreover, S1R regulates proper tau phosphorylation and axon extension that are primary events required for neuroplasticity and neurodevelopment [59]. The use of the *s1r^+25/+25^* zebrafish line will now foster in vivo studies on the developmental role of S1R in an integrated vertebrate organism.

The behavioral analyses of the *s1r^+25/+25^* larvae first revealed a hyperlocomotor response in the VMR test that appeared likely unrelated to visual or other neurosensorial modification. This phenotype trait is not shared by S1R KO mouse lines, since WT or KO mice failed to show basal hyperlocomotion compared to WT littermates, particularly when tested from 8 to 48-weeks of age and in different open-field or maze tests [44,55,56,60]. Locomotor behaviors, including walking in mice or swimming in zebrafish, are generated by networks of neurons known as central pattern generators located in the spinal cord of vertebrates. Morphological and physiological considerations on locomotor behaviors in zebrafish have been recently remarkably reviewed by Berg et al. [61]. In particular, it appears that central pattern generators involve: (i) motoneurons that activate muscle; (ii) ipsilateral excitatory interneurons that provide excitatory drive; (iii) inhibitory commissural interneurons that ensure left-right alternation; and (iv) ipsilateral inhibitory interneurons that contribute to burst termination [61]. S1R is highly expressed in motoneurons [62], and S1R activity has been shown to protect motoneurons from insults or neurodegeneration [15,63,64,65]. Furthermore, S1R is expressed in interneurons [66] and alteration of S1R activity directly impacts interneurons activity. For instance, in a mouse model of ALS shown to provoke a loss of S1R in lumbar motoneurons [63], choline acetyltransferase expression accumulated in the soma of motoneurons with a drastic diminution of efferences toward Renshaw interneurons [63]. It is therefore not surprising that a constitutive loss of S1R activity during zebrafish development rapidly led to locomotor alterations in larva.

Interestingly, the observation that the touch-escape response was unaltered in the *s1r^+25/+25^* larva, as compared to *s1r^WT^* control larva, suggested that the hyperlocomotor response was restricted to the spinal swim neuronal network, but did not affect more complex integrated networks. This was unexpected, as the touch-escape behavior was one of the first behavioral responses that allowed monitoring in vivo in zebrafish of the Ca^2+^ rise associated with single action potentials in the dendrites, soma and nucleus [67]. A massive activation of the motoneuron pool, and a differential activation of populations of hindbrain neurons, were elicited by the escape response and, as S1R activity has been shown to be an important physiological modulator of intracellular Ca^2+^ mobilization [3], a behavioral impact could be measured. However, the touch-escape behavior involves a relay of sensory information to populations of reticulospinal neurons, including the command-like Mauthner neuron, which in turn excites target neurons in the spinal cord. This connectivity implements a dynamic behavioral response that transiently interrupts ongoing swimming, directing the fish away from the threat [61]. Considering the major ethological importance of the behavior, it is likely that the neuronal network adapted to the developmental absence of the S1R intracellular modulatory effect in either motoneurons or interneurons.

Since S1R has been shown to be expressed in RGCs, PRs, RPE cells and surrounding the soma of cells in the inner nuclear layer [68], we analyzed the effect of the loss of S1R in the presence of RGCs and PRs. Notably, in *s1r^+25/+25^* larva, a significant decrease of the number of RGCs and in the size of the rods outer segments was observed. This data confirms the important role of S1R in retinal physiology. Nevertheless, our VMR experiment failed to detect any deficit, suggesting that the loss of retinal cells is not sufficient to induce physiological alteration. In order to determine if lack of S1R leads to retinal deficit, a more precise investigation measuring ERG [69] in our model would be useful. 

S1R activity is crucial at MAMs, where it simultaneously modulates the induction of ER stress pathways by releasing Bip [1] and chaperoning IRE1 [5], and (ii) mitochondrial homeostasis, as previously detailed. Koshenov et al. [46] recently provided an elegant demonstration in human neuroblastoma SH-SY5Y cells that ER stress is a physiological signal promoting S1R-induced increase in mitochondrial ATP production. The authors propose that S1R activity orchestrates Ca^2+^ leak from the ER to promote mitochondrial bioenergetics and maintain a balanced metabolism of reactive oxygen species during early stress event. The latter was induced by a limited 2-h exposure of the cells to Tunicamycin [46]. Indeed, we observed a marked deregulation of ER stress response in *s1r^+25/+25^* zebrafish line, with very decreased expression of the genes associated with the IRE1 and PERK pathways, namely, *ire1* and *perk*, but also of their effectors *xbp1s/us* and *eif2s1*/*atf4β*, respectively. These decreased levels were related to increased responses to the Tunicamycin challenge. Indeed, exaggerated increases following the stressor treatment were observed for *ire1*, *xbp1s/us*, *atf4β* and even for *atf6*, suggesting that the ATF6 pathway is mobilized to compensate for IRE1 and PERK. S1R activity has repeatedly been shown to be related to IRE1 and PERK pathways in physiopathological conditions [5,70,71]. These deregulations are coherent with a major role of S1R in the ER stress response, and more generally in the regulation between the ER stress response and mitochondrial bioenergetics. The *s1r^+25/+25^* zebrafish line will, therefore, be a valuable tool for the in vivo validation of these mechanistical hypotheses, and to better apprehend the crucial role of S1R in ER-mitochondrial communication as suggested by recent studies [46,53,71]. Finally, we demonstrated that S1R is able to modulate the expression level of BiP without affecting its mRNA. This interesting observation suggests that S1R controls the expression level of BiP at a post-translational level, highlighting a novel mode of regulation of the first orchestrator of the UPR, BiP. Other experiments are needed to decipher how the lack of S1R leads to the BiP protein deregulation and its fundamental role in the control of the UPR in basal and stressed conditions.

Analyses of the mitochondrial oxidative respiration of *s1r^+25/+25^* zebrafish line showed significant decreases, particularly in basal respiration capacity and in the respiration linked to the production of ATP. Basal respiration is the respiration used to meet the endogenous ATP demand of the cell and drive the proton leak pathway. ATP-linked respiration estimates the respiration that is used to drive mitochondrial ATP synthesis. The latter is the sum of the ATP utilization, ATP synthesis and substrate supply and oxidation. By facilitating Ca^2+^ transfer into the mitochondria, S1R activity contributes to the proper functioning of the tricarboxylic acid (TCA) cycle through facilitation of pyruvate dehydrogenase activity. The TCA cycle provides NADH cofactor to the complexes of the respiratory chain. In turn, S1R activity was shown to facilitate oxidative respiration in basal physiological conditions in brain cells [47] as well as in cardiac cells [68]. S1R activity particularly increased complex I activity, which is a NADH-dependent ubiquinone oxidoreductase, the activity of which is also Ca^2+^-dependent and directly modulated by mitochondrial calcium uniporter (MCU) activity [53,70,72]. Therefore, in zebrafish, the data confirmed that S1R activity is not a requisite to mitochondria physiology but appears necessary, as S1R invalidation resulted in a −26% decrease in basal respiration and −34% in the respiration related to the formation of ATP. Induction of ER stress by Tunicamycin resulted in significant alterations of the different parameters related to oxidative respiration, as previously observed [46,73,74] that were progressive and observed at shorter exposure times (2 h, 4 h, data not shown). Interestingly, loss of S1R abolished the decrease of basal and ATP-linked OCR after Tunicamycin treatment, suggesting that S1R was necessary for the adaptation of mitochondrial physiology to ER stress. However, how S1R interferes with the modulation of basal and ATP-linked respiration requires further investigation. Our data confirmed that S1R activity plays an important role in the physiopathological regulation of mitochondrial bioenergetics [46,53] and confirmed the potentialities of the zebrafish model for the in vivo studies of these aspects. 

## 4. Materials and Methods

### 4.1. Zebrafish and Maintenance 

The present study followed the recommendations of the ARRRIVE guidelines [75] and the European Union Directive 2010/63. The zebrafish *s1r^+25/+25^* line was a gift from Drs Jing-Ruey Joanna Yeh and Randall T. Peterson (Cardiovascular Research Center, Massachusetts General Hospital, Charlestown, MA, USA) [39]. Briefly, the fish were generated using CRISPR-cas9, creating a 25bp insertion into exon 2 of the *sigmar1* gene. Adult zebrafish were bred and maintained under standard conditions in an automated fish tank system (ZebTEC, Tecniplast, West Chester, PA, USA) at 28 °C, pH 7, conductivity around 500 mS and with a 14 h:10 h light:dark cycle. Eggs were obtained by natural spawning and maintained in E3 medium (5 mM NaCl, 0.17 mM KCl, 0.33 mM CaCl_2_, 0.33 mM MgSO_4_, 0.05% methylene blue) at 28 °C. Unfertilized embryos were discarded and the water was changed only the first day. Larvae were euthanized by an extended immersion in an ice bath for biochemical experiments. Each experimental procedure was carried out in triplicate and larvae originated from three different crosses.

### 4.2. Genotyping

For isolated homozygous *s1r^WT^* and *s1r^+25/+25^* zebrafish, genomic DNA was extracted from the tail fins at 2 months of age and the tissue was lysed in 50 mM NaOH at 95 °C for 1 h. A 237 bp region of *sigmar1* gene, encompassing the 25 bp insertion, was amplified by PCR using GoTaq Green Master Mix (Promega, Madison, WI, USA). The primers were as follows: *sigmar1,* 5′-ATAGGTCAGGATCATGAGCAGG-3′ (forward); 5′-TTATGACCTGAATGTCCACCGG-3′ (reverse). The DNA fragments were separated by electrophoresis on a 3% agarose gel and the genotype analyzed. The control amplicon should give one band of 237 bp while the mutant should give one band of 212 bp. The mutation was confirmed by Sanger sequencing.

### 4.3. Chemical Treatment

To induce ER stress, larvae were incubated at 4 dpf for 24 h with 2 µg/mL of Tunicamycin (sc-3606, Santa Cruz Biotechnology, Santa Cruz, CA, USA) diluted in 0.1% DMSO. Control larvae were treated with 0.1% DMSO diluted in E3 medium.

### 4.4. RT-PCR and Quantitative Real-Time PCR (qPCR)

Total RNA from 20 pooled whole *s1r^WT^* or *s1r^+25/+25^* larvae at 5 dpf were extracted using a Nucleospin^®^ RNA kit (Macherey-Nagel, Hoerdt, France) according to the manufacturer’s instructions. Concentration and purity were evaluated using the Agilent RNA 6000 Nano^®^ Kit (Agilent Technologies, Santa Clara, CA, USA). RNA samples (1 µg/µL) were denatured 5 min at 70 °C and reverse transcribed into cDNA, 1 h at 37 °C, using M-MLV reverse transcriptase (Promega). Primer sequences are detailed in the Supplementary Table S1. Control reactions were performed with sterile water to determine signal background and DNA contamination. The standard curve of each gene was confirmed to be in a linear range, while *zef1α* gene was used as a reference.

### 4.5. Visual Motor Response (VMR) Assay

The VMR assay quantifies the locomotor activity of the zebrafish larvae to light intensity changes. The protocol was previously described (Crouzier et al., 2021). In brief, 5 dpf larvae were transferred in a 96-well plate (#7701-1651, Whatman, Maidstone, UK) with 300 µL E3 medium and placed in a Zebrabox^®^ (ViewPoint, Lissieu, France). The locomotor behavior was monitored using an automated video-tracking device equipped with an infra-red (IR) camera under IR light illumination (Zebralab^®^, Viewpoint). The light protocol was as follow: 30 min of acclimatization in the dark (0% light intensity), then two cycles of 10-min duration light ON and 10-min duration light OFF periods. The brightness changes were immediate (<<1 s). The activity was measured in mm/s. For each larva, the mean (OFF-ON) values were calculated to account for inter and intragroup variability in locomotor response.

### 4.6. Acoustic Startle Response (ASR) Assay

The ASR assay quantifies the locomotor activity of zebrafish larvae to a sound stimulus. The ASR test was also performed in the ZebraBox^®^. Experimental conditions were similar as those used for the VMR. The protocol consisted first in acclimating larvae during 30 min in silence (35 dB ambient), followed by a 1-s stimulation with a white noise at 90 dB repeated three times with an intertrial time interval of 5 min. The amount of movement was measured for each larva. To normalize the values, baseline activity, i.e., the activity recorded during 2 min before each stimulation, was subtracted from the post stimulation activity levels.

### 4.7. Optokinetic Response (OKR) Assay

The zebrafish larvae were immersed per group of four in a Petri dish (35 mm diameter) containing 2.5% methylcellulose (#9004-65-3, Sigma Aldrich, St. Louis, MO, USA). Larvae were placed dorsal up and in a X shape, to avoid touching and interfering with each other. All measurements were done between 2:00 and 6:00 p.m. The room temperature was 28 °C and the light was off. Visual system performance of larval zebrafish was assessed using a videotracking device (VisioBox^®^, ViewPoint). Forty-six mm wide black-and-white strips were projected at 2 rpm for 1 min clockwise and then 1 min counterclockwise. Eye saccades were recorded for 2 min at 25 frames/s using a FL3-U3-32S2M 1/2.8-inch monochrome IR camera (FLEA3, FLIR). The number of eye saccades was recorded (PHIVisualize software) and data expressed as number of saccades per 2 min. 

### 4.8. Touch Response

Larvae were individually placed at one extremity of a rail (18 cm × 0.4 cm) filled with 200 µL of E3 medium. The room temperature was 28 °C and the light was on. The larva tail was briefly touched with a tip and the escape distance measured during 5 s. The same procedure was repeated three times with an intertrial time interval of 1 min to reduce stress. Data was expressed as the averaged distance swam per larva. 

### 4.9. Morphological Analyses

After prolonged exposure to ice, larvae were completely immobilized in a 2.5% methylcellulose solution contained in a petri dish. Larvae size and eye area were measured with ImageJ software v1.46 (NIH, Bethesda, MD, USA) on an image taken with a stereo microscope (Olympus, Tokyo, Japan), at ×3.2 magnification. Ear and otoliths area were measured at ×6.3 magnification.

### 4.10. Immunohistochemistry

Whole larvae were fixed in paraformaldehyde at 4 °C for 48 h, cryoprotected in 30% sucrose and mounted in O.C.T.^TM^ medium (Sakura, Tissue-Tek, Alphen aan den Rijn, The Netherlands). They were transversely sectioned in 10-µm thick slices using a cryostat (Leica, Wetzlar, Germany) at −20 °C and mounted on a glass slide. Cryosections were blocked with a solution containing 0.1% PBS/Triton X-100 and 5% horse serum for 30 min at room temperature. They were subsequently incubated overnight at 4 °C with the following primary antibodies: mouse anti-Rho4d2 (1:7000; ab98887, Abcam, Cambridge, UK), mouse anti-ZPR-1 (1:500; ab174435, Abcam) or rabbit anti-cleaved caspase-3 (Asp175) (1:500, #9661, Cell Signaling Technology, Danvers, MA, USA). After several washes, sections were incubated with the following conjugated secondary antibodies: Cy3 anti-mouse (dilution 1:800; #715-165-150, Jackson ImmunoResearch Europe Ltd., Ely, UK), Cy3 anti-rabbit (1:1000; #711-166-152, Jackson ImmunoResearch) or Alexa Fluor 488 anti-mouse (1:1000; #715-545-150, Jackson ImmunoResearch). Nuclei were counterstained with 40,6-diamidino-2 phenylendole (DAPI; 1:5000; Sigma Aldrich). The emitted fluorescence was measured using a confocal microscope (LSM880 Fastairyscan, Zeiss, Germany). 

### 4.11. Cell Counting

Cone cells, immunolabeled with Zpr-1 antibody were quantified individually and the total area of rod outer segments, immunolabeled with Rho4d2 antibody, was evaluated. Both measures were normalized to the length of the associated retina. The number of apoptotic cells was assessed by directly counting cells labeled with the anti-cleaved caspase-3 antibody per entire retina section. Ganglion cells, highlighted by DAPI counterstaining, were counted in three specific regions of the retina, consistent from one sample to another. The total number of ganglion cells from all regions was averaged per larva. The thickness of the ganglion cells layer was also quantified for each distinct region of the retina.

### 4.12. Seahorse XF Cell Mito Stress Test

The oxygen consumption rate (OCR) of 5 dpf larvae was measured with a Seahorse XFe24 extracellular flux analyzer (Agilent), according to [76]. The Seahorse temperature space was maintained at 28 °C. The larvae were placed individually in a well of a Seahorse XFe24 spheroid microplate, in 500 μL of E3 medium. A grid was placed on the larvae to maintain them at the bottom of the wells throughout the experiment. Two wells were kept empty per experiment and were considered as the “blank” condition. At 4 dpf, the larvae were treated for 24 h with tunicamycin (2 μg/mL) diluted in E3 medium and placed at 5 dpf in the measurement plate without treatment. Control larvae were treated with 0.1% DMSO. Measurements of total zebrafish OCR were performed according to the manufacturer’s instructions. Four basal analysis cycles were recorded, then five cycles recorded after administration of oligomycin (25 μM), five cycles after administration of carbonyl cyanide-p-trifluoromethoxyphenylhydrazone (FCCP) (8 μM) and nine cycles after rotenone + antimycin A (15 μM). Calculations of the different OCR parameters (nonmitochondrial respiration; basal respiration; maximal respiration; proton leak; ATP production) were done according to the Seahorse XF Cell Mito Stress Test Kit user guide (Agilent). 

### 4.13. Western Blot Analyses

Twenty 5 dpf larvae were pooled and homogenized on ice for 15 s in 100 µL of lysis buffer (Dulbecco’s Phosphate Buffered Saline, 1% NP40, 0.5% sodium deoxycholate, 0.1% sodium dodecyl sulfate, sodium orthovanadate, phenylmethylsulfonyl fluoride, PhosSTOP^TM^ (Roche, Basel, Switzerland), Complete^TM^ (Roche)). Between 20 and 40 μg of total proteins were separated on a 1.5 mm 12% running gel and 4% acrylamide stacking gel at 100 V. Proteins were transferred into a nitrocellulose membrane at 100 V for 1 h in transfer buffer and blocked with 5% nonfat milk solution for 1 h. Immunoblotting was performed in 0.1% TBS/Triton X-100 buffer pH 7.4, overnight at 4 °C, with the following primary antibodies: rabbit anti-S1R antibody (1:500, 15168-1-AP; Proteintech, Rosemont, IL, USA); rabbit anti-Chop antibody (1:1000, G6916, Sigma Aldrich); rabbit anti-Bip (1:700, SPC-180, Biosciences); rabbit anti-Eif2α (D9G8) (1:500, #3398, Cell Signaling); rabbit anti-p-Eif2α (D9G8) (1:500, #9722, Cell Signaling). After several washes, membranes were incubated with horseradish peroxidase (HRP) conjugated goat anti-rabbit secondary antibody (1:2000; ab6721, Abcam) or goat anti-mouse secondary antibody (1:2000; ab6789, Abcam) for 1 h at room temperature. The membranes were incubated with the indicated HRP detection reagent (10776189, Merck, Germany) and the bands were visualized using the Bio-Rad imaging system. Relative intensities of each band were quantified using Image Lab v6.1 software (Bio-Rad, Hercules, CA, USA) and normalized to the total protein quantity (Stain-Free^TM^, Bio-Rad). The original blots used in the Figures are presented in Supplementary Figure S3.

### 4.14. Statistical Analyses

Data were expressed as mean ± SEM. Statistical significance between groups was determined by a two-way ANOVA, with zebrafish genotype and treatment as independent factors, and/or an unpaired Student’s *t*-test (for parametric data) or a Mann-Whitney’s test (for nonparametric data). All two-way ANOVA results are detailed in the Supplementary Table S2. The levels of statistical significance considered were *p* < 0.05, *p* < 0.01 and *p* < 0.001. Statistical analyses were performed using the GraphPad Prism v7.0 software.

## Data Availability

Data is available from the authors under reasonable request.

## Supplementary Materials

The following are available online at https://www.mdpi.com/article/10.3390/ijms222011049/s1.

## Abbreviations

ALS	Amyotrophic lateral sclerosis
ASR	Acoustic startle response
ATP	Adenosine triphosphate
ATF6	Activating transcription factor 6
BDNF	Brain-derived neurotrophic factor
BIP	Glucose-regulated protein (GRP-78)
DAPI	4′,6-Diamidino-2-phenylindole
DMSO	Dimethyl sulfoxyde
dpf	Day post fertilization
ER	Endoplasmic reticulum
FCCP	Carbonyl cyanide-p-trifluoromethoxyphenylhydrazone
HRP	horseradish peroxydase
IP3R	Inositol 1,4,5-trisphosphate receptor
IRE1	Inositol-requiring enzyme 1
KO	Knockout
MAM	Mitochondria-associated ER membrane
MCU	Mitochondrial calcium uniporter
OCR	Oxygen consumption rate
OKR	Optokinetic response
PERK	Protein kinase R (PKR)-like endoplasmic reticulum kinase
qPCR	Quantitative polymerase chain reaction
S1R	σ_1_ Receptor
TCA	Tricarboxylic acid
UPR	Unfolded protein response
VMR	Visual motor response

## References

[1] Hayashi T., Su T.P. (2007). Sigma-1 receptor chaperones at the ER-mitochondrion interface regulate Ca^2+^ signaling and cell survival. Cell.

[2] Penke B., Bogár F., Fülöp L. (2017). β-Amyloid and the pathomechanisms of Alzheimer’s disease: A comprehensive view. Molecules.

[3] Hayashi T., Maurice T., Su T.P. (2000). Ca^2+^ signaling via sigma_1_-receptors: Novel regulatory mechanism affecting intracellular Ca^2+^ concentration. J. Pharmacol. Exp. Ther..

[4] Su T.P., Hayashi T., Maurice T., Buch S., Ruoho A.E. (2010). The sigma-1 receptor chaperone as an inter-organelle signaling modulator. Trends Pharmacol. Sci..

[5] Mori T., Hayashi T., Su T.P. (2012). Compromising σ_1_ receptors at the endoplasmic reticulum render cytotoxicity to physiologically relevant concentrations of dopamine in a nuclear factor-κB/Bcl-2-dependent mechanism: Potential relevance to Parkinson’s disease. J. Pharmacol. Exp. Ther..

[6] Eisner V., Picard M., Hajnóczky G. (2018). Mitochondrial dynamics in adaptive and maladaptive cellular stress responses. Nat. Cell Biol..

[7] Brailoiu G.C., Deliu E., Console-Bram L.M., Soboloff J., Abood M.E., Unterwald E.M., Brailoiu E. (2016). Cocaine inhibits store-operated Ca^2+^ entry in brain microvascular endothelial cells: Critical role for sigma-1 receptors. Biochem. J..

[8] Zhang H., Cuevas J. (2002). Sigma receptors inhibit high-voltage-activated calcium channels in rat sympathetic and parasympathetic neurons. J. Neurophysiol..

[9] Zhemkov V., Ditlev J.A., Lee W.R., Wilson M., Liou J., Rosen M.K., Bezprozvanny I. (2021). The role of sigma 1 receptor in organization of endoplasmic reticulum signaling microdomains. Elife.

[10] Su T.P., Junien J.L., Itzhak Y. (1994). Sigma receptors in the central nervous system and the periphery. Sigma Receptors.

[11] Vilner B.J., John C.S., Bowen W.D. (1995). Sigma-1 and sigma-2 receptors are expressed in a wide variety of human and rodent tumor cell lines. Cancer Res..

[12] Hayashi T., Su T. (2005). The sigma receptor: Evolution of the concept in neuropsychopharmacology. Curr. Neuropharmacol..

[13] Maurice T., Goguadze N. (2017). Sigma-1 (σ_1_) receptor in memory and neurodegenerative diseases. Handb. Exp. Pharmacol..

[14] Maurice T. (2021). Bi-phasic dose response in the preclinical and clinical developments of sigma-1 receptor ligands for the treatment of neurodegenerative disorders. Expert Opin. Drug Discov..

[15] Mancuso R., Oliván S., Rando A., Casas C., Osta R., Navarro X. (2012). Sigma-1R agonist improves motor function and motoneuron survival in ALS mice. Neurotherapeutics.

[16] Mavlyutov T.A., Epstein M.L., Verbny Y.I., Huerta M.S., Zaitoun I., Ziskind-Conhaim L., Ruoho A.E. (2013). Lack of sigma-1 receptor exacerbates ALS progression in mice. Neuroscience.

[17] Peviani M., Salvaneschi E., Bontempi L., Petese A., Manzo A., Rossi D., Salmona M., Collina S., Bigini P., Curti D. (2014). Neuroprotective effects of the Sigma-1 receptor (S1R) agonist PRE-084, in a mouse model of motor neuron disease not linked to SOD1 mutation. Neurobiol. Dis..

[18] Bhuiyan M.S., Fukunaga K. (2009). Stimulation of sigma-1 receptor signaling by dehydroepiandrosterone ameliorates pressure overload-induced hypertrophy and dysfunctions in ovariectomized rats. Expert Opin. Ther. Targets.

[19] Tagashira H., Bhuiyan S., Shioda N., Hasegawa H., Kanai H., Fukunaga K. (2010). Sigma1-receptor stimulation with fluvoxamine ameliorates transverse aortic constriction-induced myocardial hypertrophy and dysfunction in mice. Am. J. Physiol. Heart Circ. Physiol..

[20] Kibaly C., Meyer L., Patte-Mensah C., Mensah-Nyagan A.G. (2008). Biochemical and functional evidence for the control of pain mechanisms by dehydroepiandrosterone endogenously synthesized in the spinal cord. FASEB J..

[21] Gris G., Merlos M., Vela J.M., Zamanillo D., Portillo-Salido E. (2014). S1RA, a selective sigma-1 receptor antagonist, inhibits inflammatory pain in the carrageenan and complete Freund’s adjuvant models in mice. Behav. Pharmacol..

[22] Urani A., Roman F.J., Phan V.L., Su T.P., Maurice T. (2001). The antidepressant-like effect induced by sigma1-receptor agonists and neuroactive steroids in mice submitted to the forced swimming test. J. Pharmacol. Exp. Ther..

[23] Chaki S., Nakazato A., Kennis L., Nakamura M., Mackie C., Sugiura M., Vinken P., Ashton D., Langlois X., Steckler T. (2004). Anxiolytic- and antidepressant-like profile of a new CRF1 receptor antagonist, R278995/CRA0450. Eur. J. Pharmacol..

[24] Omi T., Tanimukai H., Kanayama D., Sakagami Y., Tagami S., Okochi M., Morihara T., Sato M., Yanagida K., Kitasyoji A. (2014). Fluvoxamine alleviates ER stress via induction of Sigma-1 receptor. Cell Death Dis..

[25] Romieu P., Martin-Fardon R., Bowen W.D., Maurice T. (2003). Sigma_1_ receptor-related neuroactive steroids modulate cocaine-induced reward. J. Neurosci..

[26] Maurice T., Casalino M., Lacroix M., Romieu P. (2003). Involvement of the sigma 1 receptor in the motivational effects of ethanol in mice. Pharmacol. Biochem. Behav..

[27] John C.S., Vilner B.J., Geyer B.C., Moody T., Bowen W.D. (1999). Targeting sigma receptor-binding benzamides as in vivo diagnostic and therapeutic agents for human prostate tumors. Cancer Res..

[28] Gregianin E., Pallafacchina G., Zanin S., Crippa V., Rusmini P., Poletti A., Fang M., Li Z., Diano L., Petrucci A. (2016). Loss-of-function mutations in the SIGMAR1 gene cause distal hereditary motor neuropathy by impairing ER-mitochondria tethering and Ca^2+^ signalling. Hum. Mol. Genet..

[29] Lee J.J.Y., van Karnebeek C.D.M., Drögemoller B., Shyr C., Tarailo-Graovac M., Eydoux P., Ross C.J., Wasserman W.W., Björnson B., Wu J.K. (2016). Further Validation of the SIGMAR1 c.151+1G>T mutation as cause of distal hereditary motor neuropathy. Child Neurol. Open.

[30] Al-Saif A., Al-Mohanna F., Bohlega S. (2011). A mutation in sigma-1 receptor causes juvenile amyotrophic lateral sclerosis. Ann. Neurol..

[31] Watanabe S., Ilieva H., Tamada H., Nomura H., Komine O., Endo F., Jin S., Mancias P., Kiyama H., Yamanaka K. (2016). Mitochondria-associated membrane collapse is a common pathomechanism in SIGMAR1- and SOD1-linked ALS. EMBO Mol. Med..

[32] Couly S., Goguadze N., Yasui Y., Kimura Y., Wang S.M., Sharikadze N., Wu H.E., Su T.P. (2020). Knocking Out Sigma-1 Receptors Reveals Diverse Health Problems. Cell. Mol. Neurobiol..

[33] Bai Q., Burton E.A. (2011). Zebrafish models of Tauopathy. Biochim. Biophys. Acta.

[34] Bandmann O., Burton E.A. (2010). Genetic zebrafish models of neurodegenerative diseases. Neurobiol. Dis..

[35] Das S., Rajanikant G.K. (2014). Huntington disease: Can a zebrafish trail leave more than a ripple?. Neurosci. Biobehav. Rev..

[36] Laird A.S., Mackovski N., Rinkwitz S., Becker T.S., Giacomotto J. (2016). Tissue-specific models of spinal muscular atrophy confirm a critical role of SMN in motor neurons from embryonic to adult stages. Hum. Mol. Genet..

[37] Howe K., Clark M.D., Torroja C.F., Torrance J., Berthelot C., Muffato M., Collins J.E., Humphray S., McLaren K., Matthews L. (2013). The zebrafish reference genome sequence and its relationship to the human genome. Nature.

[38] Taylor K.L., Grant N.J., Temperley N.D., Patton E.E. (2010). Small molecule screening in zebrafish: An in vivo approach to identifying new chemical tools and drug leads. Cell Commun. Signal..

[39] Rennekamp A.J., Huang X.P., Wang Y., Patel S., Lorello P.J., Cade L., Gonzales A.P., Yeh J.R., Caldarone B.J., Roth B.L. (2016). σ_1_ receptor ligands control a switch between passive and active threat responses. Nat. Chem. Biol..

[40] Hanner M., Moebius F.F., Flandorfer A., Knaus H.G., Striessnig J., Kempner E., Glossmann H. (1996). Purification, molecular cloning, and expression of the mammalian sigma1-binding site. Proc. Natl. Acad. Sci. USA.

[41] Ruoho A.E., Chu U.B., Ramachandran S., Fontanilla D., Mavlyutov T., Hajipour A.R. (2012). The ligand binding region of the sigma-1 receptor: Studies utilizing photoaffinity probes, sphingosine and N-alkylamines. Curr. Pharm. Des..

[42] Schmidt H.R., Zheng S., Gurpinar E., Koehl A., Manglik A., Kruse A.C. (2016). Crystal structure of the human σ_1_ receptor. Nature.

[43] Mavylutov T., Chen X., Guo L., Yang J. (2018). APEX2- tagging of Sigma 1-receptor indicates subcellular protein topology with cytosolic N-terminus and ER luminal C-terminus. Protein Cell.

[44] Langa F., Codony X., Tovar V., Lavado A., Giménez E., Cozar P., Cantero M., Dordal A., Hernández E., Pérez R. (2003). Generation and phenotypic analysis of sigma receptor type I (σ_1_) knockout mice. Eur. J. Neurosci..

[45] Yamashita D., Sun G.W., Cui Y., Mita S., Otsuki N., Kanzaki S., Nibu K., Ogawa K., Matsunaga T. (2015). Neuroprotective effects of cutamesine, a ligand of the sigma-1 receptor chaperone, against noise-induced hearing loss. J. Neurosci. Res..

[46] Koshenov Z., Oflaz F.E., Hirtl M., Pilic J., Bachkoenig O.A., Gottschalk B., Madreiter-Sokolowski C.T., Rost R., Malli R., Graier W.F. (2021). Sigma-1 receptor promotes mitochondrial bioenergetics by orchestrating ER Ca^2+^ leak during early ER stress. Metabolites.

[47] Hayashi T., Justinova Z., Hayashi E., Cormaci G., Mori T., Tsai S.Y., Barnes C., Goldberg S.R., Su T.P. (2010). Regulation of sigma-1 receptors and endoplasmic reticulum chaperones in the brain of methamphetamine self-administering rats. J. Pharmacol. Exp. Ther..

[48] Mitsuda T., Omi T., Tanimukai H., Sakagami Y., Tagami S., Okochi M., Kudo T., Takeda M. (2011). Sigma-1Rs are upregulated via PERK/eIF2α/ATF4 pathway and execute protective function in ER stress. Biochem. Biophys. Res. Commun..

[49] Su T.P. (2019). Non-canonical targets mediating the action of drugs of abuse: Cocaine at the sigma-1 receptor as an example. Front. Neurosci..

[50] Hedskog L., Pinho C.M., Filadi R., Rönnbäck A., Hertwig L., Wiehager B., Larssen P., Gellhaar S., Sandebring A., Westerlund M. (2013). Modulation of the endoplasmic reticulum-mitochondria interface in Alzheimer’s disease and related models. Proc. Natl. Acad. Sci. USA.

[51] Angebault C., Fauconnier J., Patergnani S., Rieusset J., Danese A., Affortit C.A., Jagodzinska J., Mégy C., Quiles M., Cazevieille C. (2018). ER-mitochondria cross-talk is regulated by the Ca^2+^ sensor NCS1 and is impaired in Wolfram syndrome. Sci. Signal..

[52] Delprat B., Crouzier L., Su T.P., Maurice T. (2020). At the crossing of ER stress and MAMs: A key role of sigma-1 receptor?. Adv. Exp. Med. Biol..

[53] Goguadze N., Zhuravliova E., Morin D., Mikeladze D., Maurice T. (2019). Sigma-1 receptor agonists induce oxidative stress in mitochondria and enhance complex i activity in physiological condition but protect against pathological oxidative stress. Neurotox. Res..

[54] Sabino V., Cottone P., Parylak S.L., Steardo L., Zorrilla E.P. (2009). Sigma-1 receptor knockout mice display a depressive-like phenotype. Behav. Brain. Res..

[55] Chevallier N., Keller E., Maurice T. (2011). Behavioural phenotyping of knockout mice for the sigma-1 (σ₁) chaperone protein revealed gender-related anxiety, depressive-like and memory alterations. J. Psychopharmacol..

[56] Eachus H., Choi M.K., Ryu S. (2021). The effects of early life stress on the brain and behaviour: Insights from zebrafish models. Front. Cell Dev. Biol..

[57] Tsai S.Y., Hayashi T., Harvey B.K., Wang Y., Wu W.W., Shen R.F., Zhang Y., Becker K.G., Hoffer B.J., Su T.P. (2009). Sigma-1 receptors regulate hippocampal dendritic spine formation via a free radical-sensitive mechanism involving Rac1xGTP pathway. Proc. Natl. Acad. Sci. USA.

[58] Ryskamp D.A., Zhemkov V., Bezprozvanny I. (2019). Mutational Analysis of Sigma-1 Receptor’s Role in Synaptic Stability. Front. Neurosci..

[59] Tsai S.Y., Chuang J.Y., Tsai M.S., Wang X.F., Xi Z.X., Hung J.J., Chang W.C., Bonci A., Su T.P. (2015). Sigma-1 receptor mediates cocaine-induced transcriptional regulation by recruiting chromatin-remodeling factors at the nuclear envelope. Proc. Natl. Acad. Sci. USA.

[60] Fontanilla D., Johannessen M., Hajipour A.R., Cozzi N.V., Jackson M.B., Ruoho A.E. (2009). The hallucinogen N,N-dimethyltryptamine (DMT) is an endogenous sigma-1 receptor regulator. Science.

[61] Berg E.M., Björnfors E.R., Pallucchi I., Picton L.D., El Manira A. (2018). Principles governing locomotion in vertebrates: Lessons from zebrafish. Front. Neural Circuits.

[62] Alonso G., Phan V., Guillemain I., Saunier M., Legrand A., Anoal M., Maurice T. (2000). Immunocytochemical localization of the sigma(1) receptor in the adult rat central nervous system. Neuroscience.

[63] Casas C., Herrando-Grabulosa M., Manzano R., Mancuso R., Osta R., Navarro X. (2013). Early presymptomatic cholinergic dysfunction in a murine model of amyotrophic lateral sclerosis. Brain Behav..

[64] Gaja-Capdevila N., Hernández N., Zamanillo D., Vela J.M., Merlos M., Navarro X., Herrando-Grabulosa M. (2021). Neuroprotective effects of sigma 1 receptor ligands on motoneuron death after spinal root injury in mice. Int. J. Mol. Sci..

[65] Naia L., Ly P., Mota S.I., Lopes C., Maranga C., Coelho P., Gershoni-Emek N., Ankarcrona M., Geva M., Hayden M.R. (2021). The Sigma-1 receptor mediates pridopidine rescue of mitochondrial function in Huntington disease models. Neurotherapeutics.

[66] Zhang B., Wang L., Chen T., Hong J., Sha S., Wang J., Xiao H., Chen L. (2017). Sigma-1 receptor deficiency reduces GABAergic inhibition in the basolateral amygdala leading to LTD impairment and depressive-like behaviors. Neuropharmacology.

[67] Fetcho J.R., Cox K.J., O’Malley D.M. (1998). Monitoring activity in neuronal populations with single-cell resolution in a behaving vertebrate. Histochem. J..

[68] Ola M.S., Moore P., El-Sherbeny A., Roon P., Agarwal N., Sarthy V.P., Casellas P., Ganapathy V., Smith S.B. (2001). Expression pattern of sigma receptor 1 mRNA and protein in mammalian retina. Mol. Brain Res..

[69] Fleisch V.C., Jametti T., Neuhauss S.C. (2008). Electroretinogram (ERG) Measurements in Larval Zebrafish. CSH Protoc..

[70] Morihara R., Yamashita T., Liu X., Nakano Y., Fukui Y., Sato K., Ohta Y., Hishikawa N., Shang J., Abe K. (2018). Protective effect of a novel sigma-1 receptor agonist is associated with reduced endoplasmic reticulum stress in stroke male mice. J. Neurosci. Res..

[71] Shenkman M., Geva M., Gershoni-Emek N., Hayden M.R., Lederkremer G.Z. (2021). Pridopidine reduces mutant huntingtin-induced endoplasmic reticulum stress by modulation of the Sigma-1 receptor. J Neurochem..

[72] Abdullah C.S., Alam S., Aishwarya R., Miriyala S., Panchatcharam M., Bhuiyan M.A.N., Peretik J.M., Orr A.W., James J., Osinska H. (2018). Cardiac dysfunction in the sigma 1 receptor knockout mouse associated with impaired mitochondrial dynamics and bioenergetics. J. Am. Heart Assoc..

[73] He J., Gong M., Wang Z., Liu D., Xie B., Luo C., Li G., Tse G., Liu T. (2021). Cardiac abnormalities after induction of endoplasmic reticulum stress are associated with mitochondrial dysfunction and connexin43 expression. Clin. Exp. Pharmacol. Physiol..

[74] Jackisch L., Murphy A.M., Kumar S., Randeva H., Tripathi G., McTernan P.G. (2020). Tunicamycin-induced endoplasmic reticulum stress mediates mitochondrial dysfunction in human adipocytes. J. Clin. Endocrinol. Metab..

[75] Kilkenny C., Browne W., Cuthill I.C., Emerson M., Altman D.G., NC3Rs Reporting Guidelines Working Group (2010). Animal research: Reporting in vivo experiments: The ARRIVE guidelines. Br. J. Pharmacol..

[76] Lee S., Lee H., Kim K.T. (2019). Optimization of experimental conditions and measurement of oxygen consumption rate (OCR) in zebrafish embryos exposed to organophosphate flame retardants (OPFRs). Ecotoxicol. Environ. Saf..

