# Supplementary material for: Sigma-1 Receptor Is Critical for Mitochondrial Activity and Unfolded Protein Response in Larval Zebrafish
Source: Int J Mol Sci. 2021 Oct 13;22(20):11049. doi: 10.3390/ijms222011049 (PMC8537383; doi:10.3390/ijms222011049)
Supplement: Supplementary file 1 [file ijms-22-11049-s001.zip › ijms-1400528-supplementary.pdf]

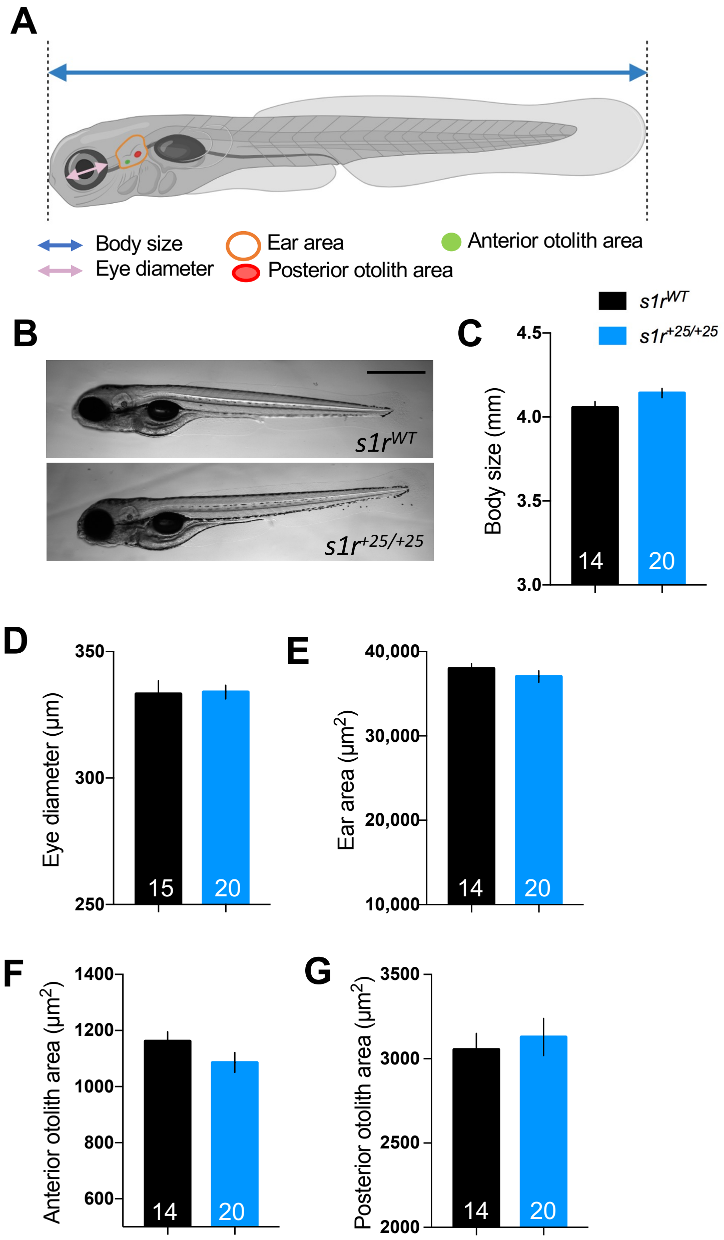

Supplemental Figure S1. Morphology of *s1r*$^{+25/+25}$ *larvae* at 5 dpf. (A) Schematic representation of the different measurements of the larva. **(B)** Images taken of wildtype or homozygous *s1r* mutant 5 dpf zebrafish. **(C)** Measurement of body length, from the mouth to the end of the tail fin and **(D)** the diameter of the eye. **(E)** The area of the ear, **(F)** anterior, **(G)** and posterior otolith were also analyzed. Scale = 1 mm. The number of animals is indicated within the columns. $p > 0.05$ for all comparisons; unpaired t-test.

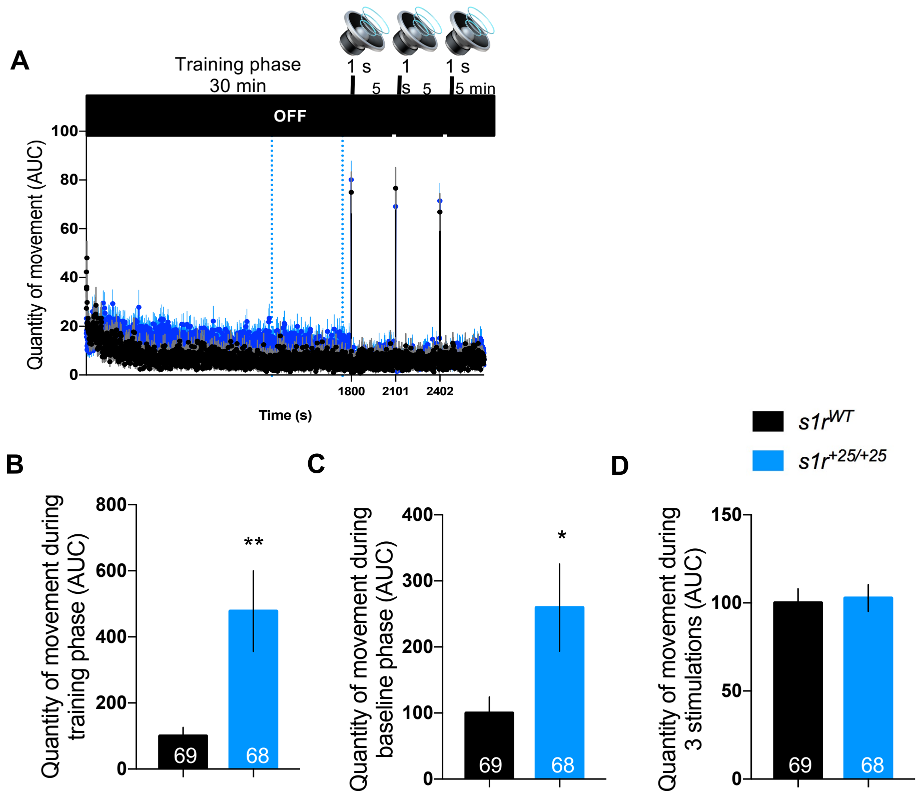

Supplemental Figure S2. Analysis of the quantity of movement by the larvae at 5 dpf during the noise cycle in the ASR test. (A) Protocol: the activity is measured for 45 min in a total dark (OFF 0%) condition, with a training phase for 30 min silently, then 3 cycles of white sounds (90 dB) of 1 s each and 5 min interspersed. **(A)** Quantity of movement per second for WT and mutant larvae according to the sound protocol. Relative quantity of movement during: **(B)** the training phase [blue dotted lines in **(A)**, between 21 and 29 min]; **(C)** the baseline phase, period of 2 min before each sound [the averaged 3 baseline phases]; **(D)** the 3 stimulations [the averaged sounds phases]. Activity were expressed as % of controls. Error bars represent ± SEM calculated from three replicas. The number of animals is indicated within the columns. * $p < 0.05$, ** $p < 0.01$; unpaired t-test.

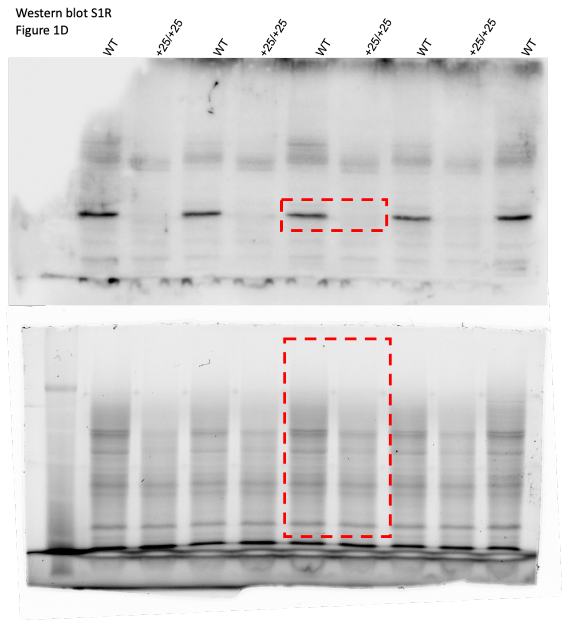

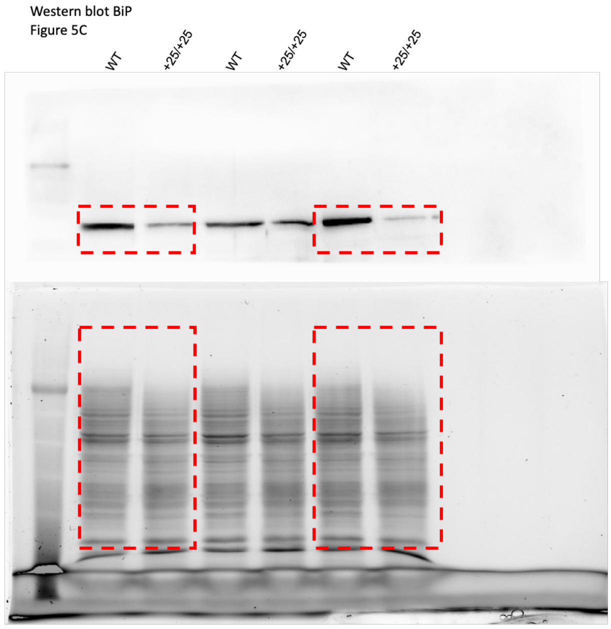

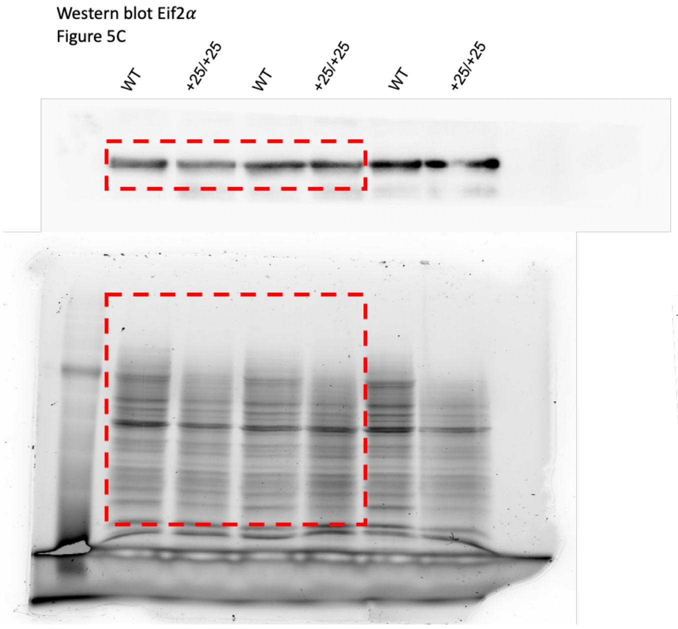

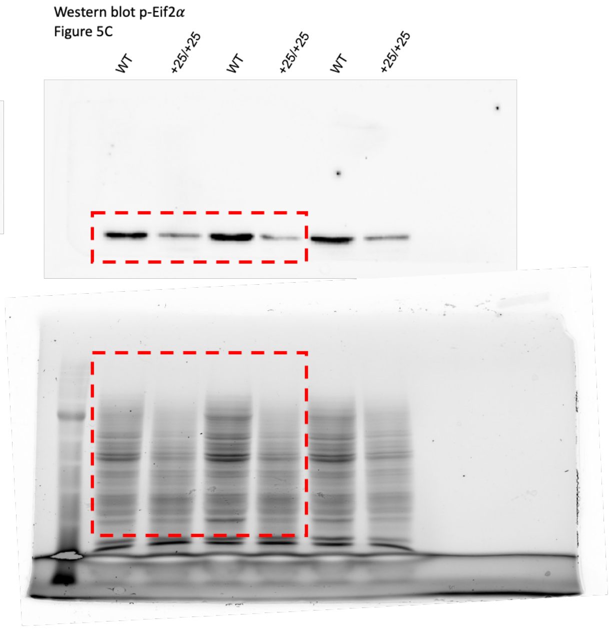

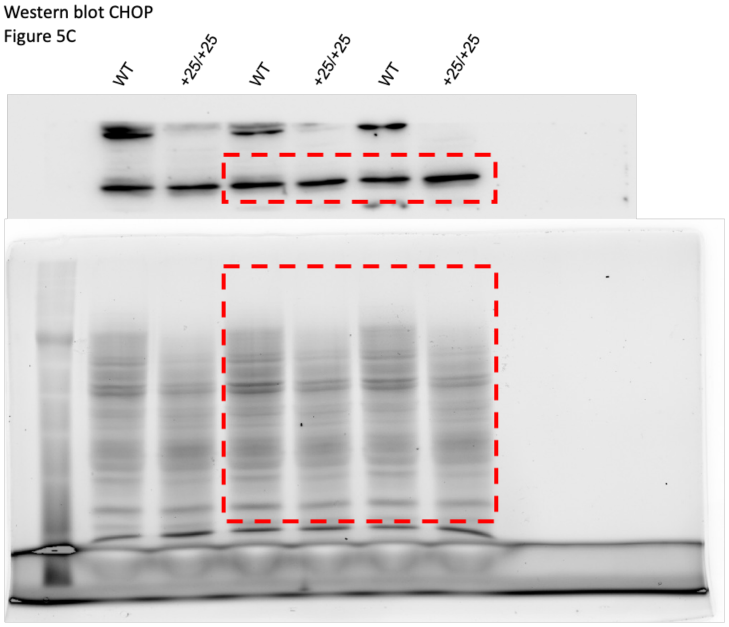

Supplemental Figure S3. Orginal blots used in Figure 1 and in Fugure 5. The hatched line box indicate the lanes presented in the article and the respective stain-free zone used for normalization.

Supplementary Table S1. List of primers used for qPCR analyses

Target genes	***Sequences***
s1r	*forward*: ATGAGCAGGCCTTCTCTAAGGT<br>*reverse*: TAGTCGTCCCCTCTTTCCATTG
bip	*forward*: AAGAGGCCGAAGAGAAGGAC<br>reverse: AGCAGCAGAGCCTCGAAATA
hsp90b1	*forward*: GGCGTTAATCTGCTATTGAG<br>*reverse*: GTCTTTGGTTTGTCCTTGTC
ire1	*forward*: TGACGTGGTGGAAGTTGGTA<br>*reverse*: ACGGATCACATTGGGATGTT
xbp1s	*forward*: TGTTGCGAGACAAGACGA<br>*reverse*: CCTGCACCTGCTGCGGACT
xbp1us	*forward*: GGGTTGGATACCTTGGAAA<br>*reverse*: AGGGCCAGGGCTGTGAGTA
perk	*forward*: TGGGCTCTGAAGAGTTCGAT<br>*reverse*: TGTCAGCCTTCTCCGTCTTT
eif2s1	*forward*: CCAAAGATGAGCAGCTGGAGA<br>*reverse*: ATCCGACACAGCCTGCTTAAA
atf4α	*forward*: CCGGGAATCATGGCAGTGTA<br>*reverse*: GAGAAGCTGCGGTATTTGCG
atf4β	*forward*: TGACCCTCTGCGGTCAATTC<br>*reverse*: ACGAATGATCTTCACCACTGTCT
atf4β	*forward*: TGACCCTCTGCGGTCAATTC<br>*reverse*: ACGAATGATCTTCACCACTGTCT
perk	*forward*: TGGGCTCTGAAGAGTTCGAT<br>*reverse*: TGTCAGCCTTCTCCGTCTTT
atf6	*forward*: CTGTGGTGAAACCTCCACCT<br>*reverse*: CATGGTGACCACAGGAGATG
chop	*forward*: CTGATTGGTGCGATGACTGC<br>*reverse*: ACTCGGGCTCCTTCTCTGAA
zef1α	*forward*: TTCTGTTACCTGGCAAAGGG<br>*reverse*: TTCAGTTTGTCCAACACCCA

Supplementary Table S2. Statistical analyses report

Figure 1.

(**C**) Mann-Whitney: $p < 0.0001$
(**E**) Mann-Whitney: $p = 0.0286$

Figure 2.

(**B**) Unpaired *t*-test: $t_{(96)} = 0.4381$, $p = 0.6623$
(**C**) Unpaired *t*-test: $t_{(96)} = 2.861$, $p = 0.0052$
(**D**) Unpaired *t*-test: $t_{(96)} = 2.504$, $p = 0.0140$
(**F**) Unpaired *t*-test: $t_{(102)} = 1.798$, $p = 0.0751$
(**G**) Unpaired *t*-test: $t_{(46)} = 0.05299$, $p = 0.958$

Figure 3.

(**A**) Unpaired *t*-test: $t_{(10)} = 9.072$, $p < 0.0001$
(**B**) Unpaired *t*-test: $t_{(10)} = 0.5687$, $p = 0.5821$
(**D**) Unpaired *t*-test: $t_{(16)} = 0.6290$, $p = 0.5382$
(**F**) Unpaired *t*-test: $t_{(10)} = 2.988$, $p = 0.0136$

Figure 4.

(**A**) Unpaired *t*-test:
bip: $t_{(19)} = 0.08225$, $p = 0.9353$
hsp90b1: $t_{(19)} = 1.912$, $p = 0.0710$
ire1: $t_{(22)} = 6.202$, $p < 0.0001$
xbp1s: $t_{(21)} = 14.24$, $p < 0.0001$
xbp1us: $t_{(22)} = 14.96$, $p < 0.0001$
perk: $t_{(21)} = 3.252$, $p = 0.00382$
eif2s1: $t_{(22)} = 8.838$, $p < 0.0001$
atf4α: $t_{(22)} = 1.941$, $p = 0.06516$
atf4β: $t_{(22)} = 2.519$, $p = 0.01954$
atf6: $t_{(22)} = 0.4258$, $p = 0.6744$
chop: $t_{(22)} = 2.421$, $p = 0.02418$

(**B**) Unpaired *t*-test:
Bip: $t_{(16)} = 3.859$, $p = 0.0014$
p-Eif2s1/Eif2s1: $t_{(15)} = 3.365$, $p = 0.0043$
Chop: $t_{(16)} = 1.146$, $p = 0.2685$

Figure 5.

(**A**) Two-way ANOVA:
s1r: $F_{(1,16)} = 536.8$, $p < 0.0001$ for the genotype, $F_{(1,16)} = 4.831$, $p = 0.043$ for the treatment, $F_{(1,16)} = 4.831$, $p = 0.043$ for the interaction
bip: $F_{(1,16)} = 4.38$, $p = 0.0527$ for the genotype, $F_{(1,16)} = 74.78$, $p < 0.0001$ for the treatment, $F_{(1,16)} = 5.577$, $p = 0.0312$ for the interaction
hsp90b1: $F_{(1,16)} = 2.868$, $p = 0.1097$ for the genotype, $F_{(1,16)} = 68,37$, $p < 0.0001$ for the treatment, $F_{(1,16)} = 1.264$, $p = 0.2775$ for the interaction
ire1: $F_{(1,16)} = 19.95$, $p = 0.0004$ for the genotype, $F_{(1,16)} = 284.4$, $p < 0.0001$ for the treatment, $F_{(1,16)} = 8.056$, $p = 0.0119$ for the interaction
xbp1s: $F_{(1,16)} = 26.81$, $p < 0.0001$ for the genotype, $F_{(1,16)} = 165.6$, $p < 0.0001$ for the treatment, $F_{(1,16)} = 9.391$, $p = 0.0074$ for the interaction
xbp1us: $F_{(1,16)} = 37.74$, $p < 0.0001$ for the genotype, $F_{(1,16)} = 54.33$, $p < 0.0001$ for the treatment, $F_{(1,16)} = 7.201$, $p = 0.0163$ for the interaction

perk: $F_{(1,16)}$ = 4.382, p = 0.0526 for the genotype, $F_{(1,16)}$ = 99.24, p < 0.001 for the treatment, $F_{(1,16)}$ = 1.330, p = 0.2658 for the interaction
eif2s1: $F_{(1,16)}$ = 2.669, p = 0.1218 for the genotype, $F_{(1,16)}$ = 2.801, p = 0.1136 for the treatment, $F_{(1,16)}$ = 1.073, p = 0.3156 for the interaction
atf4α: $F_{(1,16)}$ = 0.03893, p = 0.8461 for the genotype, $F_{(1,16)}$ = 29.86, p < 0.0001 for the treatment, $F_{(1,16)}$ = 2.411, p = 0.1401 for the interaction
atf4β: $F_{(1,16)}$ = 24.85, p = 0.0001 for the genotype, $F_{(1,16)}$ = 4.621, p = 0.0472 for the treatment, $F_{(1,16)}$ = 2.925, p = 0.1065 for the interaction
atf6: $F_{(1,16)}$ = 23.53, p = 0.0002 for the genotype, $F_{(1,16)}$ = 107.6, p < 0.0001 for the treatment, $F_{(1,16)}$ = 6.983, p = 0.0177 for the interaction
chop: $F_{(1,16)}$ = 6.624, p = 0.0204 for the genotype, $F_{(1,16)}$ = 1202, p < 0.0001 for the treatment, $F_{(1,16)}$ = 0.2313, p = 0.6371 for the interaction

Figure 6.

(**B**) Unpaired *t*-test: $t_{(52)}$ = 3.279, p = 0.0019
(**C**) Unpaired *t*-test: $t_{(52)}$ = 3.352, p = 0.00158
(**D**) Unpaired *t*-test: $t_{(52)}$ = 0.5627, p = 0.5761
(**E**) Unpaired *t*-test: $t_{(52)}$ = 1.257, p = 0.2144
(**F**) Unpaired *t*-test: $t_{(52)}$ = 2.559, p = 0.0135

Figure 7.

(**B**) Two-way ANOVA: $F_{(1,68)}$ = 13.57, p = 0.0005 for the genotype, $F_{(1,68)}$ = 21.23, p < 0.0001 for the treatment, $F_{(1,68)}$ = 17.730, p = 0.0070 for the interaction
(**C**) Two-way ANOVA: $F_{(1,68)}$ = 13.05, p = 0.0006 for the genotype, $F_{(1,68)}$ = 24.21, p < 0.0001 for the treatment, $F_{(1,68)}$ = 9.639, p = 0.028 for the interaction
(**D**) Two-way ANOVA: $F_{(1,68)}$ = 5.490, p = 0.0221 for the genotype, $F_{(1,68)}$ = 2.578, p = 0.1130 for the treatment, $F_{(1,68)}$ = 0.006574, p = 0.9356 for the interaction
(**E**) Two-way ANOVA: $F_{(1,68)}$ = 5.153, p = 0.0264 for the genotype, $F_{(1,68)}$ = 28.24, p < 0.0001 for the treatment, $F_{(1,68)}$ = 0.3237, p = 0.5713 for the interaction
(**F**) Two-way ANOVA: $F_{(1,68)}$ = 5.265, p = 0.0249 for the genotype, $F_{(1,68)}$ = 31.85, p < 0.0001 for the treatment, $F_{(1,68)}$ = 0.002341, p = 0.9616 for the interaction

Supplementary Figure S1.

(**C**) Unpaired *t*-test: $t_{(32)}$ = 1.94, p = 0.0612
(**D**) Unpaired *t*-test: $t_{(33)}$ = 0.1337, p = 0.8945
(E) Unpaired *t*-test: $t_{(32)}$ = 1.000, p = 0.3248
(**F**) Unpaired *t*-test: $t_{(32)}$ = 1.473, p = 0.1506
(**G**) Unpaired *t*-test: $t_{(32)}$ = 0.481, p = 0.6338

Supplementary Figure S3.

(**B**) Unpaired *t*-test: $t_{(135)}$ = 3.034, p = 0.0029
(**C**) Unpaired *t*-test: $t_{(135)}$ = 2.286, p = 0.0238
(**D**) Unpaired *t*-test: $t_{(135)}$ = 0.2516, p = 0.8017
